# Thermal stratification drives movement of a coastal apex predator

**DOI:** 10.1038/s41598-017-00576-z

**Published:** 2017-04-03

**Authors:** Eneko Aspillaga, Frederic Bartumeus, Richard M. Starr, Àngel López-Sanz, Cristina Linares, David Díaz, Joaquim Garrabou, Mikel Zabala, Bernat Hereu

**Affiliations:** 1 Departament de Biologia Evolutiva, Ecologia i Ciències Ambientals, Universitat de Barcelona, 08028 Barcelona, Catalonia Spain; 2 Centre d’Estudis Avançats de Blanes (CEAB-CSIC), 17300 Blanes, Catalonia Spain; 3 Centre de Recerca Ecològica i Aplicacions Forestals (CREAF), 08193 Cerdanyola del Vallès, Catalonia Spain; 4 Institució Català de Recerca i Estudis Avançats (ICREA), 08010 Barcelona, Catalonia Spain; 5 Moss Landing Marine Laboratories, 95139 Moss Landing, CA United States of America; 6 Institut de Ciències del Mar (ICM-CSIC), 08003 Barcelona, Catalonia Spain; 7 Centre Oceanogràfic de les Balears (COB-IEO), 07015 Palma de Mallorca, Balearic Islands Spain; 8 Aix-Marseille University, Mediterranean Institute of Oceanography (MIO), Université de Toulon, CNRS/IRD, Campus de Luminy 13288 Marseille Cedex 9, France

## Abstract

A characterization of the thermal ecology of fishes is needed to better understand changes in ecosystems and species distributions arising from global warming. The movement of wild animals during changing environmental conditions provides essential information to help predict the future thermal response of large marine predators. We used acoustic telemetry to monitor the vertical movement activity of the common dentex (*Dentex dentex*), a Mediterranean coastal predator, in relation to the oscillations of the seasonal thermocline during two summer periods in the Medes Islands marine reserve (NW Mediterranean Sea). During the summer stratification period, the common dentex presented a clear preference for the warm suprathermoclinal layer, and adjusted their vertical movements following the depth changes of the thermocline. The same preference was also observed during the night, when fish were less active. Due to this behaviour, we hypothesize that inter-annual thermal oscillations and the predicted lengthening of summer conditions will have a significant positive impact on the metabolic efficiency, activity levels, and population dynamics of this species, particularly in its northern limit of distribution. These changes in the dynamics of an ecosystem’s keystone predator might cascade down to lower trophic levels, potentially re-defining the coastal fish communities of the future.

## Introduction

Temperature is a key environmental factor that, through profound physiological effects, influences the fitness and survival of ectothermic organisms^1–3^. Ectothermic organisms are usually adapted to live within a limited range of temperatures, which includes a thermal optimum that maximizes their physiological performance^4^. When conditions move away from this optimum, an organism experiences reduced growth, reproduction, foraging, or competitiveness^5, 6^. Temperature shifts from the preferred range will thus greatly affect the dynamics of a species, altering the relative abundances of a population and changing the horizontal and vertical distribution of individuals^7–10^. In the case of ecosystem keystone species such as apex predators, community-changing herbivores, or structure-forming species, temperature-driven changes in abundance may cause ripple-effects in other levels of the food web, changing the structure and functioning of the ecosystem^11, 12^. In the face of global climate change, a great deal of research effort is being expended to characterize the thermal ecology of marine organisms, as it is necessary to understand the trends in ecosystems arising from the warming of the global ocean^6, 13^.

The ability to move makes fishes much more resistant to environmental change than less mobile or sessile benthic species^14^. As with many other mobile animals, marine fishes can readily exploit thermal gradients to regulate their body temperature and increase their metabolic efficiency^15, 16^. Movement is thus a good behavioural response with which to infer the thermal ecology of a fish species. Controlled laboratory experiments have shown that fish move across thermal gradients to attain a preferred temperature^17, 18^, and have allowed the researchers to investigate the response of an individual’s internal temperature to a fluctuating environment^16^. However, laboratory experiments are not feasible for large marine predators, and hence studies in the wild using acoustic telemetry and bio-logging technologies are a much more practical approach to study the thermal preference of these animals^10, 17, 19^.

Within the complexity of oceanographic conditions, the thermocline is a prominent structure. Thermoclines are hydrographical structures caused by large temperature gradients and that generate a significant segregation of resources. Thermoclines set up a heterogeneous thermal environment wherein mobile organisms have developed behavioural responses according to the trade-off between their physiological requirements and energy demands. For instance, many oceanic predators need to maintain warm body temperatures in order to sustain their foraging activity, but often their preys concentrate in cold deep waters. In order to exploit those food resources, several tuna species^15, 20, 21^ as well as the ocean sunfish^22^ rewarm during relatively long periods in the suprathermoclinal layer before performing short excursions below the thermocline to forage. Less attention has been paid to this kind of behaviours in coastal predators^17^, despite their key roles in shaping coastal communities and their relevance as indicators of good ecosystem conservation status^23–25^.

In this study we focused on the common dentex, *Dentex dentex* (L. 1758), one of the main coastal apex predators in the Mediterranean Sea and an important fishery resource for both artisanal and recreational fisheries^26–28^. The common dentex is present along the Atlantic and Mediterranean coasts, but its populations are more abundant in central and southern Mediterranean and rare in the Northern Mediterranean Sea^29, 30^. A global decrease in fishery landings of common dentex has been reported by FAO during the last three decades^30^, reason why it is classified as ‘vulnerable’ by the International Union for the Conservation of Nature (IUCN) in the Red List of Threatened Species^31^. However, in several Mediterranean sectors its abundance seems to be increasing, as shown by an increase in fishery landings in several Spanish ports^32^, and the fast recovery of its populations in marine protected areas^25^. The common dentex inhabits infra- and circa-littoral rocky bottoms and seagrass meadows, and is more abundant at depths between 15–30 m^33, 34^. The usual depth distribution of adult individuals coincides with the depth range at which the seasonal thermocline establishes between May and October in the NW Mediterranean Sea^35^. Summer conditions in the Mediterranean Sea are characterized by high water column stability and high temperatures, resulting in a strong stratification of the water column. However, this thermocline is known to display strong vertical oscillations in short time periods, such as a few hours or days, which are mainly driven by the wind and movement of water masses, and are also dependent on local hydrographic conditions caused by coastal orientation and bathymetry^35^.

**Figure 1:**
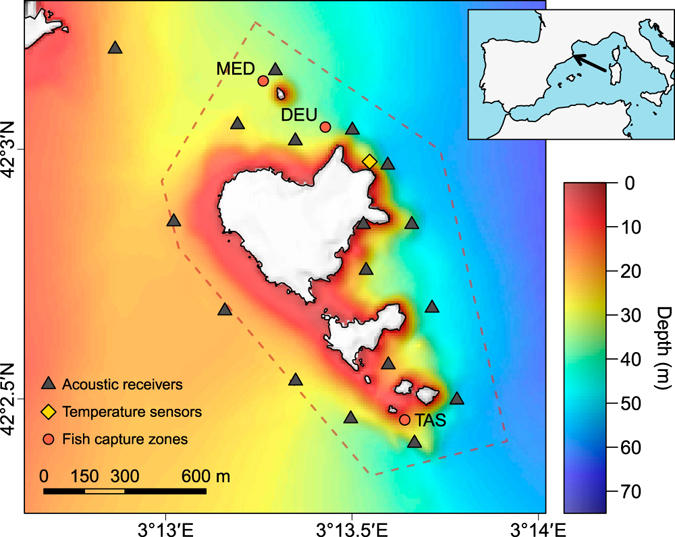
Bathymetric map of the study site and the location of acoustic receivers and temperature sensors. The approximate locations where the common dentex individuals were captured are also shown (see Table 1). *In situ* temperature sensors were placed every 5 m (between 5–40 m) in the marked location. Map was created using R^60^ version 3.3.1 (https://cran.r-project.org). The topographic base map (1:5.000), DEM and bathymetry are freely accessible through the Cartographic and Geologic Institute of Catalonia (www.icgc.cat) under Creative Commons Attribution License (CC BY 4.0).

We monitored the vertical movements of the common dentex using acoustic telemetry and characterized the thermal environment using *in situ* temperature loggers during two consecutive summers in the Medes Island marine protected area (NW Mediterranean Sea, Fig. 1). Our objective was to describe the thermal preference of the common dentex by analysing its vertical movements and activity patterns during stratified and non-stratified hydrographical periods. Describing the thermal ecology of this iconic apex predator will help us to predict their population dynamics during future warm water periods and the probable cascade effects on coastal marine ecosystems.

## Results

### Thermal regime and depth of the thermocline

**Figure 2:**
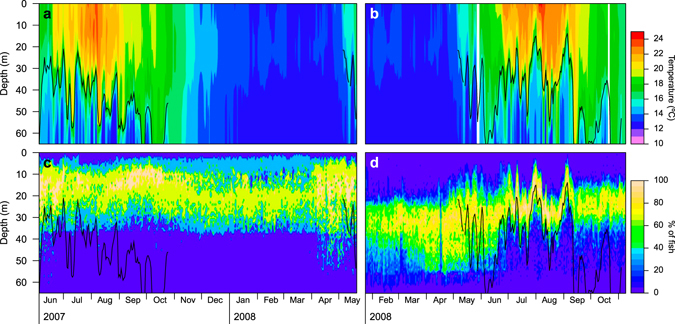
Daily temperature profiles (**a,b**) and vertical distributions of common dentex individuals (**c,d**). The panel in the left (**c**) corresponds to the set of individuals captured in May-June 2007 (n = 3), and the panel in right (**d**) to the set of individuals captured in December 2007-January 2008 (n = 9). Solid lines represent the daily mean depth of the thermocline.

**Figure 3:**
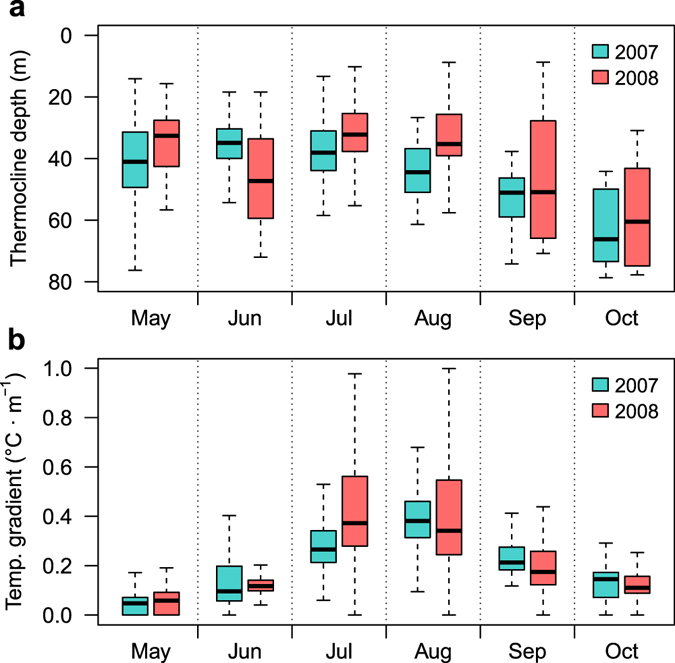
Hourly thermocline depth (**a**) and temperature gradient (**b**) values, separated by month and year. The black line near the middle and the lower and upper box boundaries represent the median and the first and third quartiles of values, respectively. Ends of the whiskers represent values at 1.5 times the interquartile range of the box.

Surface temperatures ranged from a minimum of 12.4 °C in winter (March 2008) to maximum temperatures of 23.9 °C (August 2007) and 24.6 °C (August 2008) in summer (Fig. 2a,b). The stratification of the water column started in early May and became stronger as summer progressed, with the temperature gradient increasing in strength in July and August (Fig. 3). The temperature gradient relaxed at the end of summer (September), becoming weaker and deeper, until a complete breakdown at the end of October. Shortly after that, the water column was well mixed with a temperature of about 17 °C, before displaying a progressive cooling until the minimum winter temperature.

During the maximum stratification period, with surface temperatures above 19 °C (between mid-June and mid-September), significant oscillations of the thermocline were observed in both 2007 and 2008, but there were clear differences between years (Fig. 2). The summer of 2007 presented shallower thermocline positions than the summer of 2008 (Fig. 3a). The shallowest thermocline depths in 2007 were recorded in July, during which thermocline depths shallower than 15 m occurred on only two days. By contrast, in 2008 shallow thermoclines were registered during the entire summer (July, August and September), and observations of the thermocline shallower than 15 m occurred in 14 different days. Moreover, the maximum temperature gradients observed in July and August 2008 were much more elevated than the gradients observed in the same period in 2007 (Fig. 3b).

### Vertical movement activity patterns

**Figure 4:**
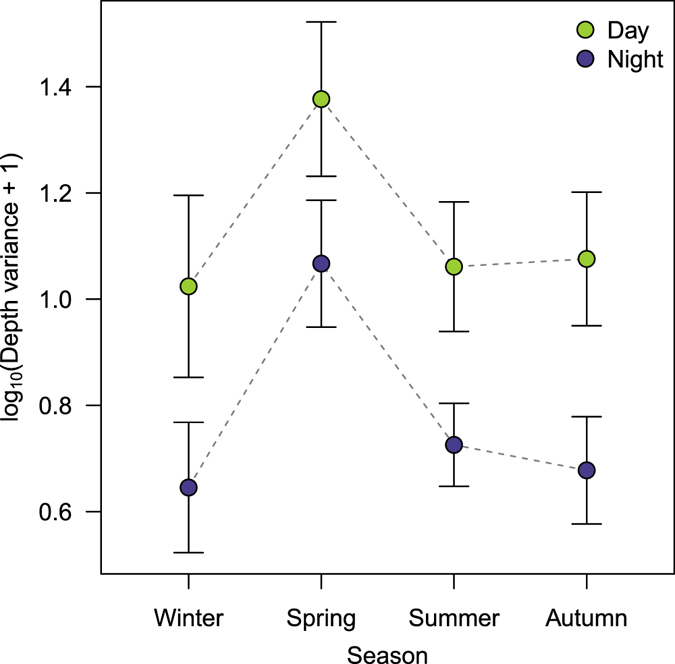
Effects of the day/night cycle and the season on the vertical movement activity of common dentex. Vertical movement activity is quantified as the variance of depth (after a logarithmic transformation). Filled circles and error bars represent the mean values predicted by the linear mixed effects model and the 95% confidence intervals, respectively.

The total length of tagged individuals ranged between 42 and 65 cm (Table 1), with no differences in size between the first set of individuals (n = 3), tagged in May-June 2007, and the second set (n = 9), tagged in December 2007-January 2008 (Kruskal-Wallis test, χ^2^ = 2.203, df = 1, p = 0.138). However, the individuals from the first set utilized shallower depths (5–30 m) than the individuals from the second set (20–45 m) (Table 1 and Fig. 2a). The linear mixed-effects model used to test the differences between vertical movements (depth fluctuations for each day/night period) revealed a significant day-night and seasonal effect (Table 2). Overall, the vertical movement activity was greater during the day than during the night, and the seasonal pattern was marked by a higher vertical movement activity in spring compared to the rest of the seasons, which did not differ (Fig. 4).

**Table 1:** Summary of the information and detections of tagged common dentex individuals.

ID	Length (cm)	Capture site	Capture date	Mean depth ± SD (m)	Total detections	Detections during the summer period (No. of 5 min intervals)		Deep excursions (% of summer detections)	
						Day	Night	Day	Night
18	63	TAS	2007-05-24	16.2 ± 4.8	64,419	11,035	5,010	0.02	0.04
43	63	TAS	2007-06-03	8.7 ± 4.5	53,049	9,436	5,017	0.01	0.00
44	59	MED	2007-06-03	23.7 ± 4.8	86,551	12,142	8,840	0.27	0.06
32	55	TAS	2007-12-02	29.7 ± 8.7	51,402	7,901	6,438	1.73	0.99
33	61	TAS	2007-12-01	29.1 ± 7.8	46,308	6,792	5,138	3.99	1.52
34	47	TAS	2007-12-01	20.9 ± 5.9	16,971	4,159	3,414	0.41	0.09
35	46	TAS	2008-01-22	26.2 ± 7.6	48,140	5,700	5,162	1.95	0.19
37	53	DEU	2007-12-02	30.5 ± 6.8	40,478	2,043	2,360	1.47	0.85
39	42	TAS	2008-01-22	25.6 ± 4.7	35,693	4,412	3,167	2.04	0.47
42	62	TAS	2007-12-01	33.9 ± 9.8	53,804	10,365	7,531	8.28	3.24
45	59	DEU	2007-12-01	37.0 ± 9.2	45,218	6,786	2,315	8.38	2.51
50	65	TAS	2007-12-01	24.0 ± 4.5	62,724	11,661	5,712	0.99	1.79

Deep excursions refer to the percentage of detections happening below the lower thermocline limit during the summer period (see Methods section).

**Table 2:** Results of the linear mixed effects model testing the effect of the day/night period and the season on vertical movement activity levels.

Level	Estimate	SE	DF	t-value	p-value
(Intercept)	1.061	0.087	6,518	12.179	<0.001**
Day/Night period					
Night	−0.410	0.041	6,518	−10.023	<0.001**
Season					
Spring	0.354	0.064	6,518	5.553	<0.001**
Summer	0.025	0.043	6,518	0.585	0.558
Autumn	0.032	0.043	6,518	0.734	0.463
Day/Night period × Season					
Night:Spring	0.073	0.022	6,518	3.400	0.001**
Night:Summer	0.046	0.021	6,518	2.183	0.020*
Night:Autumn	−0.015	0.024	6,518	−0.657	0.511

### Effect of the thermocline on fish depth

**Figure 5:**
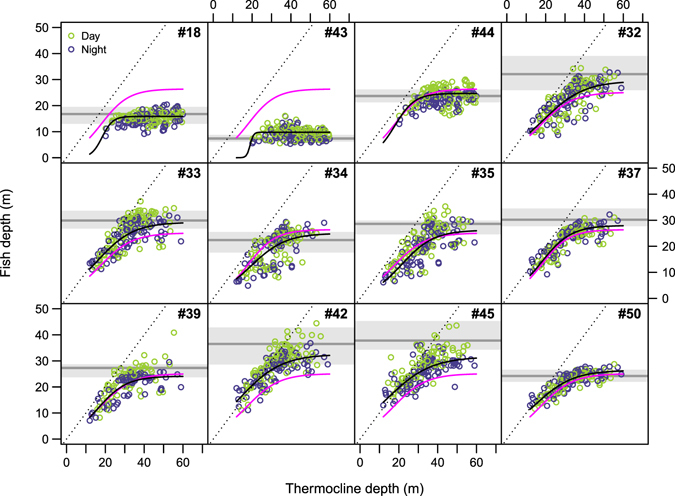
Relationship between mean fish depths and mean thermocline depths during the summer season, factorized by day/night periods, for each tagged fish (n = 12). Solid black lines represent the logistic models adjusted for each individual, and the magenta line is the mean prediction for the population. Black dotted lines highlight pure linear (1:1) relationships. Grey line and box in the bottom represent the median and the first and third quartiles, respectively, of the depths measured for each fish outside the summer period.

Tagged common dentex exhibited a characteristic pattern of vertical movements during the summer 2008, in which the depth of most of fish oscillated rapidly over periods of a few days (Fig. 2d). This pattern was not directly observable from tagged fish in the summer 2007 (Fig. 2c). A logistic mixed effects model explained the relationship between the observed depth patterns and the depth of the thermocline (Fig. 5 and Table 3). This model demonstrated a non-linear relationship between the average depths of tagged fish and the thermocline depth. When the thermocline was shallower than the depth-range used by each fish (20–45 m), the depth of the thermocline correlated positively and linearly with the mean fish depth. This relationship broke down and became asymptotic when the thermocline sank below the preferred depth range. Indeed, the individual asymptote values estimated by the model fell within the depth-range that each fish inhabited outside the summer season (Fig. 5). The effect of the day/night period was significant only in the estimation of one of the parameters of the model, the asymptote, but it had a relatively small effect (Table 3), thus indicating that individuals followed similar movement patterns above the thermocline indistinctively of the time of the day.

**Table 3:** Results of the non-linear mixed effects models testing the effect of the depth of the thermocline and the day/night period on mean fish depths.

Parameter	Estimate	S.E.	D.F.	t-value	p-value
Asym					
(Intercept)	26.39	2.10	2042	12.55	<0.001**
Night	−1.28	0.65	2042	−1.98	0.048*
x_0_					
(Intercept)	18.18	0.50	2042	36.02	<0.001**
Night	−0.73	0.69	2042	−1.07	0.287
k					
(Intercept)	6.99	0.97	2042	7.18	<0.001**
Night	1.31	0.69	2042	1.90	0.057

The fitted values for each parameter defining the logistic curve and their significance are shown: Asym = maximum value of the curve; x0 = x-value of the inflexion point; k = steepness of the curve.

Despite this average relationship, several punctual excursions below the lower limit of the thermocline were observed during the summer period (Table 1). The amount of detections below the thermocline was low (<2%) for most of fishes, excepting for three individuals, #33, #42, and #45, which presented higher percentages. Overall, the percentage of detections below the thermocline was significantly higher during the daytime than during the night (paired samples t-test: t = 2.49; df = 11, p = 0.015).

## Discussion

During the study period we observed the typical seasonal thermal cycle for the NW Mediterranean Sea, which is characterized by a mixed phase followed by a thermal stratification period^35^. Although this seasonal pattern repeats every year, there were inter-annual differences with respect to the maximum and minimum temperatures and the duration and magnitude of the stratification^35, 36^. During our study, the thermocline underwent several transient but recurring oscillations in the course of the stratification period. These oscillations included changes of up to 20 m in the depth of the thermocline and 10 °C in the temperature at certain depths. Our observations were similar to those described by Bensoussan *et al*.^35^, who indicated that thermocline oscillations represent an important fraction of the annual thermal variability despite their proportionately low duration (2.1 ± 0.8 days on average). Bensoussan *et al*.^35^ also reported that the relative variability of the temperature in summer in the study area increases with depth, reaching a maximum variability at depths between 25 and 40 m. At those depths, benthic communities are exposed to an average daily temperature variation of around 2 °C (with maximum values around 10 °C), and a weekly variation of around 7 °C (with maximum values of 11.5 °C). The swimming depth of the common dentex was strongly influenced by the temperature variations caused by the thermal structure of the water column, as shown by the relationship between the depth of the thermocline and the depths of tagged fish (Fig. 5). Transient thermocline rising events were related to upward displacements of individuals from the specific depths that they utilized outside the stratification period or during deeper thermocline events. Thus, common dentex demonstrated a clear preference for the suprathermoclinal warm water over the colder water below the thermocline.

Our main hypothesis is that a physiological optimization strategy was behind the observed behaviour of the common dentex. Temperature strongly affects the basal metabolic rate in fishes, restricting or enhancing physiological and behavioural processes^2^. Some studies on the effect of thermal gradients on fish have found that individuals spend most of their time (~75%) within ±2 °C of their preferred temperature, which coincides with the optimal physiological temperature that maximizes growth^37^. It has been also described that fish swimming speed or endurance is enhanced to a peak by an optimum temperature and is reduced when the temperature decreases or increases towards the tolerance limits^38–40^. The maximum swimming speed is a limiting factor for the foraging activity, and for this reason many oceanic predators must warm their body temperature in order to be able to hunt in cold deep waters^15, 41^. Thus, we hypothesize that the common dentex selects its thermal niche, presumably to approach the optimal temperature for growth and/or foraging in order to increase an individual’s net energy gain.

An alternative hypothesis would be that the observed movement behaviour of the common dentex was driven by indirect effects such as food distribution. The common dentex is a diurnal predator^42^, which primarily forages on other fishes (about 74% of prey items), and to a lesser degree on cephalopods and crustaceans^26, 43^. Consequently, foraging events were the most likely cause of the high vertical activity levels that we observed during the day (Fig. 4), while low activities during the night might be related to resting behaviour. Similar diel activity patterns have been observed in other coastal species^42, 44^ including other predators^17^. Regarding the relationship between the thermocline depth and the vertical distribution of individuals, our model indicated that the diel cycle had a negligible effect. This implies that a presumable concentration of prey items in the suprathermoclinal layer during the summer is not enough to explain the vertical movements of the common dentex. If that were the case, we would expect to observe a relaxation of the depth restriction imposed by the thermocline during the night, with individuals returning to their preferred depths to rest, similar to what has been described for the dogfish by Sims *et al*.^17^. On the contrary, most of the excursions below the thermocline were observed during the day, and were very probably related to punctual foraging events. Therefore, our results indicate that the observed vertical distributions of individuals during summer were more probably caused by a direct selection of the preferred temperature-range, rather than by an irregular distribution of preys items above and below the thermocline.

Our approach to estimate activity patterns could discern between day and night activity periods, but it was not adequate to resolve seasonal activity patterns. The common dentex resided within the study site throughout the year, facing two different thermal conditions (summer and winter) that we would expect to have a significant effect on fish physiology and activity. Cabled video observatories have already described a seasonal activity rhythm for the common dentex, which showed higher occurrences between August and October, indicating a change in its horizontal activity pattern coinciding with high water temperatures^45^. Similarly, Abdelkader and Ktari^46^ described an increase in the food intake of common dentex between April and May, coinciding with the timing of the spawning, and a decrease in consumption from September to February. These movement and feeding patterns are thought to be related to seasonal variations on the abundance of their prey items, mainly coastal fishes such as sparids, labrids and picarels (*Spicara maena*)^26^, which tipically are more abundant in summer than in winter. Interestingly, the common dentex has shown an unsual ability to withstand long periods without food, which seems to be an adaptation to cope with unfavourable periods^47^, such as the low prey availability and low temperatures during winter. However, our analysis did not show significant differences between the vertical activities of summer and winter. Nevertheless, it was able to detect an increase of the activity during spring, very probably related to the spawning, which has been described to occur between April and May^26^. During this period, the common dentex is thought to perform excursions to deep rocky outcrops, where it aggregates to spawn^26, 30^. Characterizing spawning movements and periods is key for designing effective conservation measures for emblematic fish species^48, 49^, and thus they should be studied more carefully in the future.

Changes in the distribution of individuals associated with seasonal and inter-annual environmental fluctuations provide insights into how populations may shift under global climate change. The current global change is driving not only a steady increase of global water temperatures, but also a lengthening of the summer period^50^. For instance, the thermal stratification has increased in the NW Mediterranean Sea during the last three decades, which has already lengthened the duration of yearly summer conditions by a ~40%^50^. Consequently, warm water fish species are being positively affected, leveraging the higher presence of favourable conditions that enhance both their metabolic and foraging efficiency, thus improving their survival rate and reproductive success^51, 52^. These kinds of environmental fluctuations are proposed as the cause of the inter-annual fluctuations in the capture rates of many important demersal fishery resources^53^, including the common dentex^30, 34^. The redistribution of preferred temperature ranges is generating changes in both the horizontal and vertical distributions of coastal fish assemblages^7, 8^. For instance, recent local data suggest that the common dentex might be expanding in the northern Mediterranean^25, 32^. Our results, by confirming the preference of this species for warm water, provides valuable information on the ecology of the species and a mechanistic understanding to the mentioned population fluctuations and expansion.

In this study, we used acoustic telemetry to provide some valuable insights on the thermal ecology of the common dentex and the possible future trends of its populations. The common dentex and other apex predators are keystone species in marine food webs, and are often used as indicators of the structure and functioning of ecosystems^23, 24^. Also, the abundances of species occupying high trophic levels shape biological communities through top-down trophic effects^54, 55^, although the extent of the impact is still under debate^56^. Climate change will differentially affect different species, depending on their physiological and behavioural traits^6, 12^, and as a result generate unpredictable effects that will interact with other perturbations such as overfishing. To make reliable predictions of future biological assemblages and main ecological trends, it is necessary to understand the mechanisms underpinning the responses of different species to climate change.

## Methods

### Acoustic telemetry study

Information about movements from 12 *D. dentex* individuals was collected from an acoustic telemetry study carried out in the Medes Islands MPA (Catalonia, NW Mediterranean Sea) between 2007 and 2008. Fish were captured by jigging hook-and-line fishing gear from a boat and tagged with V13P-1H acoustic transmitters (VEMCO, Nova Scotia; dimensions: 48 × 13 mm; power output: 153 dB; weight in water: 6.5 g), which were implanted in the peritoneal cavity using a standard surgical procedure^57, 58^. The tagging protocol followed the guidelines provided by the Ministry of Agriculture, Livestock, Fisheries and Food of the Catalan Government (decree 214/1997), and was approved by the Committee on the Ethics of Animal Experimentation of the University of Barcelona. The Department of Environment of the Catalan Government granted permissions for fishing, operating and releasing the animals in the Medes Islands Marine Reserve. All surgery was performed under 2-phenoxyethanol anaesthesia, and all efforts were made to minimize suffering. The sex of individuals could not be determined due to the lack of sexual dimorphism in this species. Transmitters were equipped with a pressure sensor and were programmed to emit signals with a random delay between 80 and 180 s. Movements of tagged individuals were monitored by a network of 17 acoustic receivers placed around the study area (Fig. 1). Signal range-tests were performed in the area and revealed an average detection range of 150 m around the receivers (see Aspillaga *et al*.^44^ for more details). Individuals were divided into two sets depending on their capture date (May-June 2007: n = 3; December 2007-January 2008: n = 9). All the analyses were restricted to a different time period for each set (Jun/4 2007 to May/21 2008, and Jan/1 2008 to Nov/11 2008, respectively), corresponding to the period in which all the individuals in the set were simultaneously tracked.

### Temperature data

Hourly measures of *in situ* temperature were provided by the T-MedNet network (http://www.t-mednet.org). Temperature was registered by autonomous sensors (HOBO Water Temp Pro v2) placed in rocky ledges at depth intervals of 5 m (from 5 to 40 m depth) at one location of the study site (Fig. 1). Additional temperature data, corresponding to manually-operated sensors for one sampling station situated at 2.5 nautical miles offshore the eastern side of the islands, was provided by J. Pascual (http://meteolestartit.cat). At that station, temperature profiles were generated every 2–3 days using a CTD that recorded measures every meter, from the surface to a depth of 90 m.

### Data analysis

The thermocline depth was calculated from hourly temperature profiles. A four parameter logistic regression was fitted to each profile, and the mean depth of the thermocline was then determined as the depth at which the first derivate of the model presented its maximum value (as in McKinzie *et al*.^59^). The upper and lower limits of the thermocline were also determined from the peaks in the second derivate of the model. The strength of the thermocline was then calculated as the temperature gradient (°C · m^−1^) between its upper and lower limits. In order to calculate the depth of the thermocline when it was below the depth-range of the autonomous sensors (40 m), the *in situ* temperature profiles were complemented with an interpolation of the manual CTD casts taken between 40 and 80 m depth. Thermocline depth was only calculated for the profiles where the total temperature difference between the surface and the deepest measures was higher than 3 °C.

Acoustic telemetry data was pooled in 5 min intervals and the mean depth was calculated for each common dentex individual, in order to remove duplicated detections in different receivers and to homogenize the data distribution along time. These intervals were then classified into consecutive day/night periods, defined by local sunset and sunrise time provided by the NOAA Solar Calculator (www.esrl.noaa.gov/gmd/grad/solcalc/) for the coordinates of the Medes Islands (42°03′N 3°13′E, WGS84), and the mean depths and variances were calculated for each period. Variance of the depth was used as an approximation to fish vertical activity, assuming that the range of vertical movements is bigger when fish are active (e.g. when foraging) than when they are resting. In order to detect diel and seasonal patterns of fish activity, a linear mixed-effects model was applied to the variance data after applying a logarithmic transformation, considering the day/night period and the season as fixed factors and the fish tag number as a random factor. To test the effect of the thermocline depth on the fish depth, only data corresponding to the summer was used, as it was the only season in which a well-developed thermocline was present. A non-linear mixed-effects model was applied to test this relationship, where the mean depth of the thermocline and day/night period were considered the primary and secondary covariates, respectively, and the fish tag number as a random factor. All the data was managed and analysed in R^60^, and mixed effects models were fitted using the ‘nlme’ package^61^. Performances of the models were visually inspected in residual distribution and residual vs. fitted values plots.
